# Prediction of Early Response to Chemotherapy in Lung Cancer by Using Diffusion-Weighted MR Imaging

**DOI:** 10.1155/2014/135841

**Published:** 2014-02-12

**Authors:** Jing Yu, Weidong Li, Zhang Zhang, Tielian Yu, Dong Li

**Affiliations:** ^1^Department of Radiology, Tianjin Medical University General Hospital, No. 154 Anshan Road, Heping District, Tianjin 300052, China; ^2^Department of Radiology, Affiliated Hospital of Xuzhou Medical College, No. 99, Huaihaixilu, Quanshan District, Xuzhou 221002, China

## Abstract

*Purpose*. To determine whether change of apparent diffusion coefficient (ADC) value could predict early response to chemotherapy in lung cancer. *Materials and Methods.* Twenty-five patients with advanced non-small cell lung cancer underwent chest MR imaging including DWI before and at the end of the first cycle of chemotherapy. The tumor's mean ADC value and diameters on MR images were calculated and compared. The grouping reference was based on serial CT scans according to Response Evaluation Criteria in Solid Tumors. Logistic regression was applied to assess treatment response prediction ability of ADC value and diameters. *Results.* The change of ADC value in partial response group was higher than that in stable disease group (*P* = 0.004). ROC curve showed that ADC value could predict treatment response with 100% sensitivity, 64.71% specificity, 57.14% positive predictive value, 100% negative predictive value, and 82.7% accuracy. The area under the curve for combination of ADC value and longest diameter change was higher than any parameter alone (*P* ≤ 0.01). *Conclusions.* The change of ADC value may be a sensitive indicator to predict early response to chemotherapy in lung cancer. Prediction ability could be improved by combining the change of ADC value and longest diameter.

## 1. Introduction

Lung cancer is the most common and highly lethal cancer worldwide with poor prognosis [1]. About 75% of patients are diagnosed at advanced stage since there is no specific recognized symptom at early stage [2]. Surgery alone is not the appropriate treatment for those patients at terminal stage [3, 4]. A large meta-analysis pooled 4,584 patients suggested that the adjuvant chemotherapy had a 5.4% improvement of 5-year survival rate in non-small cell lung cancer [5].

Assessing the early response to chemotherapy in lung cancer is crucial because optimized chemotherapy regimen needs individualization to gain a preferable outcome and to avoid toxic effect and unnecessary expenditure. Currently, computed tomography (CT) and magnetic resonance (MR) imaging are the regular methods to monitor the tumor changes in size to evaluate the effect of chemotherapy [6]. However, response assessment with these morphologic imaging has limitations in reliable differentiation of residual tumor tissue from necrotic tumor or fibrotic scar. Moreover, the tumor change in size, which lags behind the biological and molecular changes, may be not an early and sensitive indicator [7].

MR diffusion-weighted imaging (DWI) reflects the differences in the Brownian motion of water molecules between tissues [8]. As a surrogate marker of tissue cellularity by observing water mobility within the tumor, the apparent diffusion coefficient (ADC) can be used to distinguish the highly cellular tumor from normal tissue or necrotic regions [8–10]. It has been used to differentiate pulmonary malignant tumors from solid benign lesions or to stage lung cancer [11, 12]. Therefore, the change of ADC value may be used to monitor the treatment response which manifested as change in cellularity of the tumor [8, 13]. Previous studies have demonstrated that ADC values can be used as an indicator to evaluate the tumor response in tumor from many organs [13–20]. The purpose of this study is to observe the change of ADC value after chemotherapy and to determine the ability of the change of ADC value to predict treatment response in lung cancer at the early stage of chemotherapy.

## 2. Materials and Methods

### 2.1. Patients

**Figure 1:**
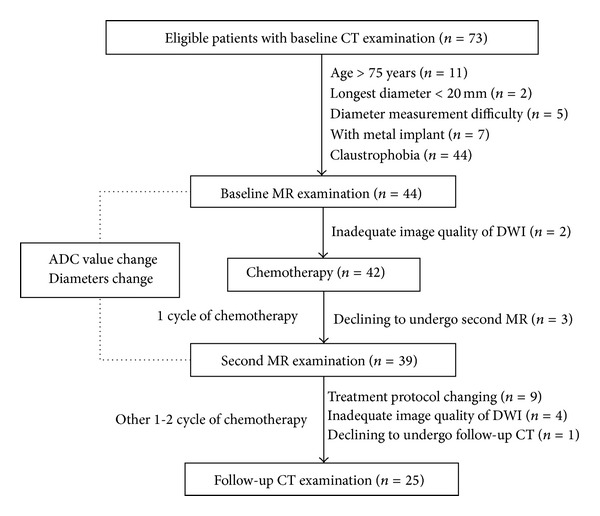
The patient selection and imaging flow chart. Baseline and follow-up CT are used for grouping.

Research ethics committee approval and patient written informed consent were obtained. All the patients participated in the study were diagnosed as non-small cell lung cancers histologically. They were scheduled for chemotherapy and with no history of previous chemotherapy or other anticancer treatments. The patient selection was shown in the diagram (Figure 1). Finally, from 2010-12 to 2012-07, 25 patients (17 male, 8 female; median age: 61.4 ± 8.0 years) with advanced non-small cell lung cancers were consecutively enrolled. There were 2 patients with multiple lung lesions. Only the largest lung lesion was included in the MR image analysis. 14 squamous cell carcinomas, 9 adenocarcinomas, and 2 adenosquamous carcinomas were confirmed histologically. The initial staging protocol was evaluated by CT, MR, SPECT, and PET-CT if available. There were 23 stage III tumors and 2 stage IV tumor.

All patients underwent concurrent chemotherapy which consisted of gemcitabine or vinorelbine on days 1 and 8 and platinum-based pharmaceutical (75 mg/m^2^) for first 2 or 3 days in each 21-day cycle. The posttherapy MR imaging was performed at the end of the first cycle of chemotherapy. Patients remained on these treatment protocols until disease progression was detected. 

### 2.2. MR

All patients accepted MR imaging one week before and after 1 cycle of chemotherapy (Figure 1). All the MR examinations were performed on a 3-T superconducting magnet (HDx; General Electric Medical Systems, Milwaukee, Wis) following the same scan protocol. Respiratory and electrocardiographically gated T2-weighted fast relaxation fast spin echo images with fat suppression images were obtained. The parameters were as follows: repetition time/echo time, 8,000~8,571 ms/86~96 ms; matrix size, 256 × 160; field of view, 42 cm; number of excitations, 2; slice thickness, 4 mm; gap, 1 mm. Electrocardiographically gated T1-weighted dual inversion recovery fast spin echo images were also obtained with the following parameters: repetition time/echo time, 1,120–1,760 ms/4.1–6.2 ms; matrix size, 256 × 160; field of view, 42 cm; number of excitations, 1; slice thickness, 4 mm; gap, 1 mm. These images were inspected initially to define locations of the pulmonary lesions for the DWI.

DWI were acquired using a respiratory gated single-shot echo-planar imaging sequence and array spatial sensitivity encoding technique with *b* values of 0 and 1000 s/mm^2^. The parameters were as follows: repetition time/echo time, 5,000–9,230 ms/55 ms; matrix size, 256 × 160; field of view, 42 cm; number of excitations, 4; slice thickness, 4 mm; gap, 1 mm; *R* factor, 2; slice-select, phase-encoding, and frequency-encoding directions. The total MR imaging acquisition time in this study was about 15 minutes.

### 2.3. Image Analysis

All MR images were transferred to a workstation (AW 4.3; GE Healthcare, Milwaukee, Wis) and analyzed by two experienced radiologists (A and B, 20 and 11 years experience in reading chest imaging, resp.), who were blinded to the therapeutic response and other data of patients. The following parameters were measured and recorded:, lesion number, location, size, and mean ADC value. This procedure was performed by the two radiologists then the mean values were calculated.

The lesion size was reflected by tumor longest and shortest diameter (in perpendicular angle) measured with a caliper tool on axial T2-weighted images. The lesion location was observed on the T2- and T1-weighted images. Meanwhile, T2- and T1-weighted images were used as a slice selection reference for ADC value measurement.

**Figure 2:**
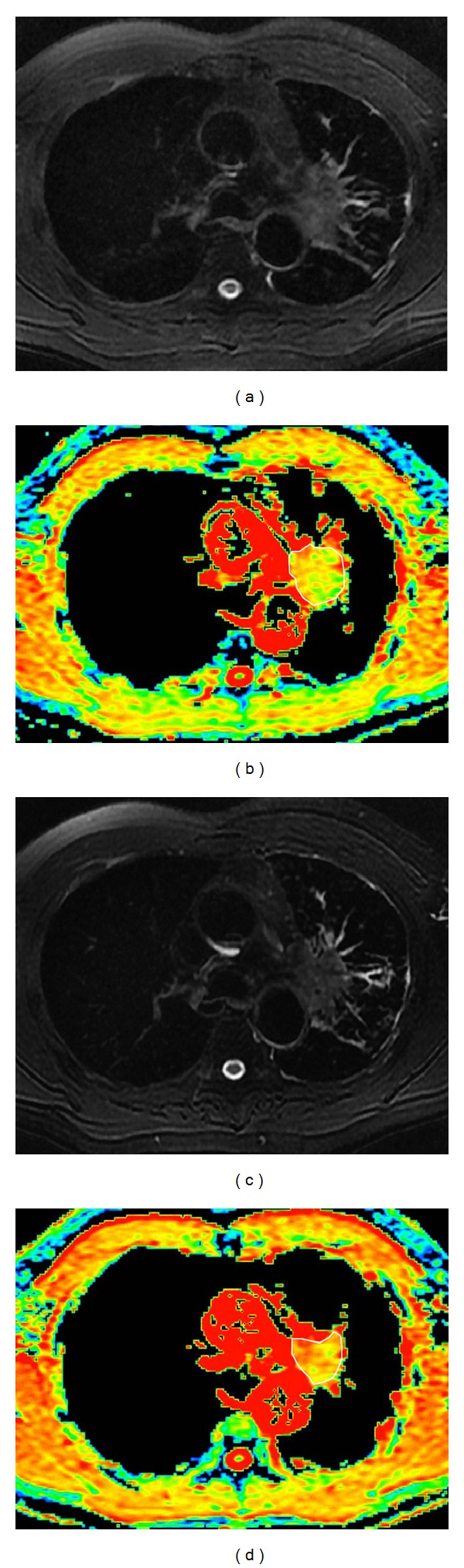
Graphs in 54-year-old man with squamous cell carcinoma lung cancer belong to partial response group. (a, c) T2-weighted with fat suppression images and apparent diffusion coefficient (ADC) maps (b, d) obtained before (a, b) and after the first cycle (c, d) of chemotherapy. From the T2-weighted with fat suppression images, no significant decrease in tumor size was detected (a, c) after the first cycle of chemotherapy, while the ADC value increased from 1.36 × 10^−3^ mm^2^/s to 2.16 × 10^−3^ mm^2^/s (b, d).

During the MR image analysis, DWI reconstructed images with *b* = 1000 s/mm^2^ were evaluated. The ADC map of each DWI image was produced on a pixel-by-pixel basis. An axial slice showing the largest tumor size corresponding to T2- and T1-weighted images was chosen. A polygonal region of interest was drawn manually encompassing the entire area of the target lesion on the ADC map (Figure 2). The mean ADC values were calculated.

### 2.4. CT Imaging

All patients underwent contrast enhanced CT scans before and after 2-3 cycles of chemotherapy conventionally (Figure 1) [21]. The images were obtained by using a 64-detector row CT (GE, light speed VCT XT) with a 64 × 0.625 mm collimation, 120 kVp, 250 mA, and 500 msec gantry rotation time in a spiral mode. The contrast enhanced CT images were acquired about 30 seconds after contrast material (Omnipaque 350, 90 mL) administration. The images were obtained in the transverse plane and then reconstructed by 2.5 mm section thickness and 2.5 mm section interval. One radiologist (C, 3 years experience in reading chest imaging), who was blinded to the results of MR images and other data of patients, measured the longest diameter of each tumor on the mediastinal window of CT images. Furthermore, the short diameters of lymph nodes (if lesions ≥15 mm in short axis) will be recorded and included in the sum of lesions in calculation of the tumor response [22]. At last, the sum of the longest tumor diameters and shortest diameters of lymph nodes was calculated.

### 2.5. PR and SD Groups

According to Response Evaluation Criteria in Solid Tumors (RECIST, version 1.1) [22, 23], the responses to chemotherapy in lung cancer were classified by B: (1) complete response: disappearance of all target lesions and reduction of any pathological lymph nodes (<10 mm in short diameter); (2) partial response (PR): at least 30% decrease in the sum of diameters of target lesions, taking as reference the baseline sum diameters; (3) stable disease (SD): neither sufficient shrinkage to qualify for PR nor sufficient increase to qualify for progressive disease; (4) progressive disease: at least 20% increase in the sum of diameters of target lesions, taking as reference the smallest sum on the study. Furthermore, a 5 mm absolute increase in sum of the target disease is needed. Based on the revised RECIST 1.1, the short diameters of lymph nodes (short diameter >15 mm) on axial CT images were included in the sum of target lesions in calculation of tumor response [23].

### 2.6. Statistical Analysis

Statistical analysis was performed using SPSS 17.0. Independent and paired student *t*-tests were used to analyze the difference of ADC value and tumor diameters between different groups or time periods. The frequencies of patient and tumor characteristics between PR and SD groups were tested by using *X* ^2^ test. To combine the changes of ADC value and diameter, a logistic regression model that allows the discrimination between PR and SD groups was employed. Receiver operator characteristic curves were generated to establish the cutoff value of the change of ADC value in order to differentiate PR lesion from SD lesion. A *P* value less than 0.05 was considered significant.

## 3. Results

In the current study, there were 8 and 17 patients in PR and SD group, respectively. No patient belonged to complete response or progressive disease group. The patient and tumor demographics between PR and SD groups was shown (Table 1).

Both longest and shortest diameter of the tumor had significant statistical differences before and after chemotherapy regardless in the SD or PR group (Table 2). The posttherapy ADC value was higher than pretherapy ADC value (*P* < 0.001) (Table 2).

**Figure 3:**
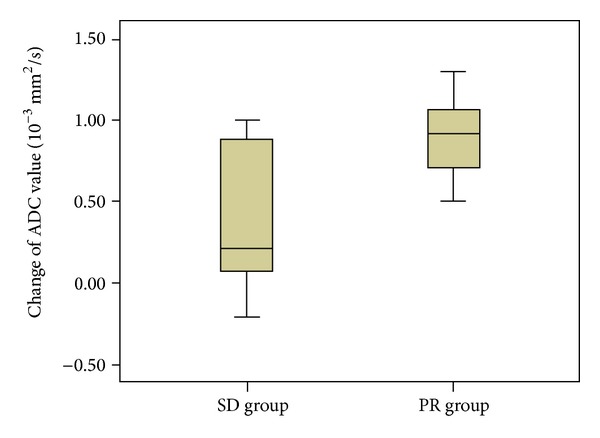
The box plot showed that the change of apparent diffusion coefficient (ADC) value in partial response (PR) group were statistically significantly greater than that in stable disease (SD) group.

When all tumors were divided into the PR and SD groups according to the mentioned criterion, it was noticed that the changes of tumor diameters on T2 images had significant difference between the SD and PR groups (*P* = 0.007 for longest diameter; *P* = 0.045 for shortest diameter) (Table 1). The pretherapy ADC value had no significant difference between the PR group and SD group (*P* = 0.517) (Table 1). The change of ADC value was statistically significantly higher in PR group compared with that in SD group (Table 1, Figure 3).

**Figure 4:**
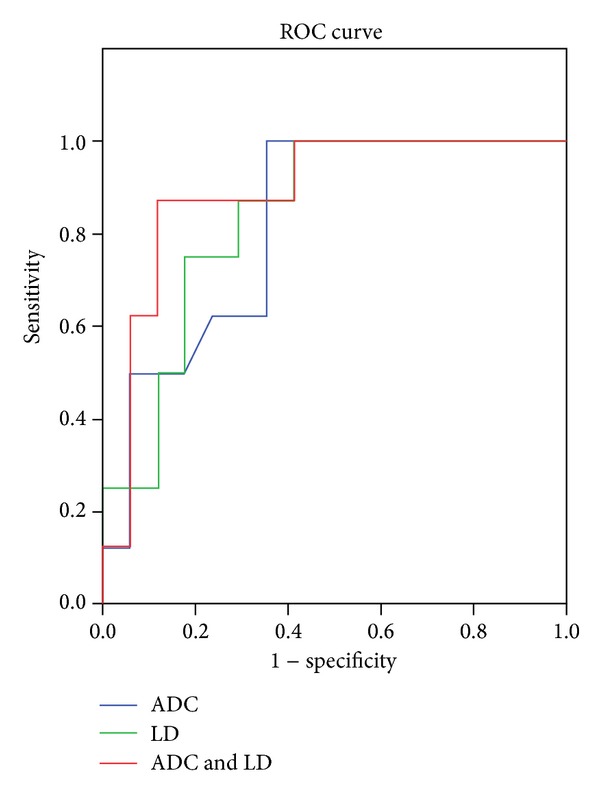
Compared with the change of apparent diffusion coefficient (ADC) value or tumor diameters alone, the combination of the change of ADC value and longest diameter (ADC & LD) had a higher area under the curve than any other alone for evaluating lung cancer treatment response (0.890, 0.827, and 0.838 for the combination, the change of ADC value, and longest diameter (LD), resp.).

The logistic regression model and receiver operator characteristic curve analysis showed that combination of the change of longest diameter and ADC value had a higher area under curve than any other parameter alone for evaluating treatment response in lung cancer (*P* < 0.01, Figure 4). When we used the change of ADC value for differentiating the PR lesion from the SD lesion, the best cutoff value was 0.41 × 10^−3^ mm^2^/s, the overall sensitivity, specificity, positive predictive value, and negative predictive value were 100%, 64.71%, 57.14%, and 100%, respectively, and the area under the receiver operator characteristic curve was 0.827.

## 4. Discussion

The chemotherapy response was usually observed after 2 cycles of chemotherapy according to tumor size change by radiographies, CT, or standard MR, rarely by functional imaging, dynamic or diffusion weighted MR imaging, or PET-CT, for example, [3, 6, 21, 24]. This study investigated whether the change of ADC value and diameters after chemotherapy could be used to evaluate early treatment response in lung cancer.

The ADC value had significant increase after 1 cycle of chemotherapy compared with baseline, especially in the PR group (Figure 3). This result was in agreement with the results of previous studies of both lung cancer and other cancers [17, 24–27]. However, the current result contradicted with the result of rectal cancer research on the decrease trend of ADC values 2–4 weeks after chemotherapy. Chemotherapy-induced fibrosis might be a contributor to decrease of ADC value [28]. The difference was probably caused by disparity in fibrosis appearance and progression. The increase of ADC value was related to necrosis and reduced cell density histologically [29], while the decrease of ADC value was relevant to cytotoxic edema and fibrosis on histology [28].

From the current results, the changes of ADC value were significant between the PR and SD groups (Table 1), which was in agreement with the previous DWI study of lung cancer treatment response evaluation after chemotherapy [24, 26], even when a different b value was used in the previous study [27]. Therefore, the noninvasive DWI could be potentially used to early predict and monitor lung cancer response to chemotherapy (Figure 2).

This study further demonstrated that the combination of longest diameter and ADC value change had a higher diagnostic ability than any other parameter alone for evaluating treatment response in lung cancer (*P* < 0.01, Figure 4). The receiver operator characteristic curve showed that the combination of longest diameter change and ADC value change adds additional value for a single parameter alone to predict treatment response. The cutoff value of ADC change could predict response to chemotherapy in lung cancer with 100% sensitivity, 64.71% specificity, 57.14% positive predictive value, 100% negative predictive value, and 82.7% accuracy.

Comparing with CT, the MR imaging had two benefits to evaluate tumor response: first, the DWI had the potential to evaluate early treatment response from the tumor inner structure change before the morphological change; second, by combining the change of ADC value and tumor diameter, the treatment response could be predicted with a high sensitivity and moderate specificity by using MR imaging only. For the small cell lung cancer, the tumor diameter change may be significant even in the early stage of chemotherapy. But for the less sensitive non-small cell lung cancer, especially on target therapy, there may not be apparent anatomical changes initially even if the chemotherapeutic regime was appropriate [29]. Therefore, tumor size evaluation alone had a smaller area under the curve than the combination of functional and anatomical assessment. The change of ADC value might have the potential to monitor and early predict lung cancer treatment response to chemotherapy. Furthermore, the diagnostic ability increased when combined with the change of ADC value and longest diameter. In our study, MR images at baseline and the end of 1 cycle of chemotherapy not only showed the tumor volume change on T2-weighted images, but also provided the change of ADC value on DWI. Even through MR imaging could not be regarded as the most appropriate and a “one stop” examination method to predict treatment response; it indeed provided precious information to CT.

There were several limitations of this study. Firstly, the sample number in our study was relatively small especially the pathological subtypes. Secondly, the tumor volume, pathologic types, and chemotherapy regimens were nonuniform, which may affect the treatment response of tumor. At last, the interval between the start of chemotherapy and treatment response evaluation by DWI was relatively long. One week interval may make the advantage of ADC value change prominent.

## 5. Conclusions

Our data suggested that the change of ADC value may be a sensitive indicator to predict early response to chemotherapy in lung cancer. Prediction ability could be improved by combining the change of ADC value and longest diameter.

## Figures and Tables

**Table 1:** Patient and tumor characteristics between the PR and SD groups.

Characteristics	PR (*n* = 8)	SD (*n* = 17)	*P*
Age (years)	66 (56–69)	59 (56–66)	0.587
Female/male	1/7	7/10	0.205
Smoker	4	9	0.891
Lesion location			0.389
Right lobe	3	11	
Left lobe	5	6	
Histological subtype			0.089
Squamous cell carcinomas	7	7	
Adenocarcinomas	1	8	
Adenosquamous carcinomas	0	2	
Stage			0.456
III A	3	8	
III B	5	7	
IV	0	2	
ADC at baseline (10^−3^ mm^2^/s)	1.26 ± 0.17	1.20 ± 0.20	0.517
Change of longest diameter (cm)	1.76 ± 0.92	0.61 ± 0.88	0.007
Change of shortest diameter (cm)	1.11 ± 0.11	0.37 ± 0.66	0.045
Change of ADC value (10^−3^ mm^2^/s)	0.90 ± 0.25	0.37 ± 0.42	0.004

ADC: apparent diffusion coefficient; PR: partial response; SD: stable disease.

**Table 2:** Comparison of ADC values and different diameters before and after chemotherapy.

Parameters	Pretherapy	Posttherapy	P
ADC (10^−3^ mm^2^/s)	1.22 ± 0.19	1.76 ± 0.47	0.000
Longest diameter (cm)	5.81 ± 2.18	4.83 ± 2.11	0.000
Shortest diameter (cm)	4.36 ± 1.65	3.75 ± 1.84	0.002

ADC: apparent diffusion coefficient.
